# P-2126. De novo belatacept-based immunosuppression is not associated with increased risk for invasive fungal infection in kidney transplant recipients

**DOI:** 10.1093/ofid/ofaf695.2290

**Published:** 2026-01-11

**Authors:** Emily Eichenberger, Geeta Karadkhele, Christian P Larsen

**Affiliations:** Emory School of Medicine, Atlanta, Georgia; Emory University School of Medicine, Atlanta, Georgia, Atlanta, Georgia; Emory University School of Medicine, Atlanta, Georgia

## Abstract

**Background:**

Belatacept is a selective co-stimulation blocker associated with improved long-term outcomes in kidney transplant recipients (KTR). The impact of belatacept on risk of invasive fungal infection (IFI) in KTR remains unclear, with existing data limited to small case series and case reports.

**Table:**
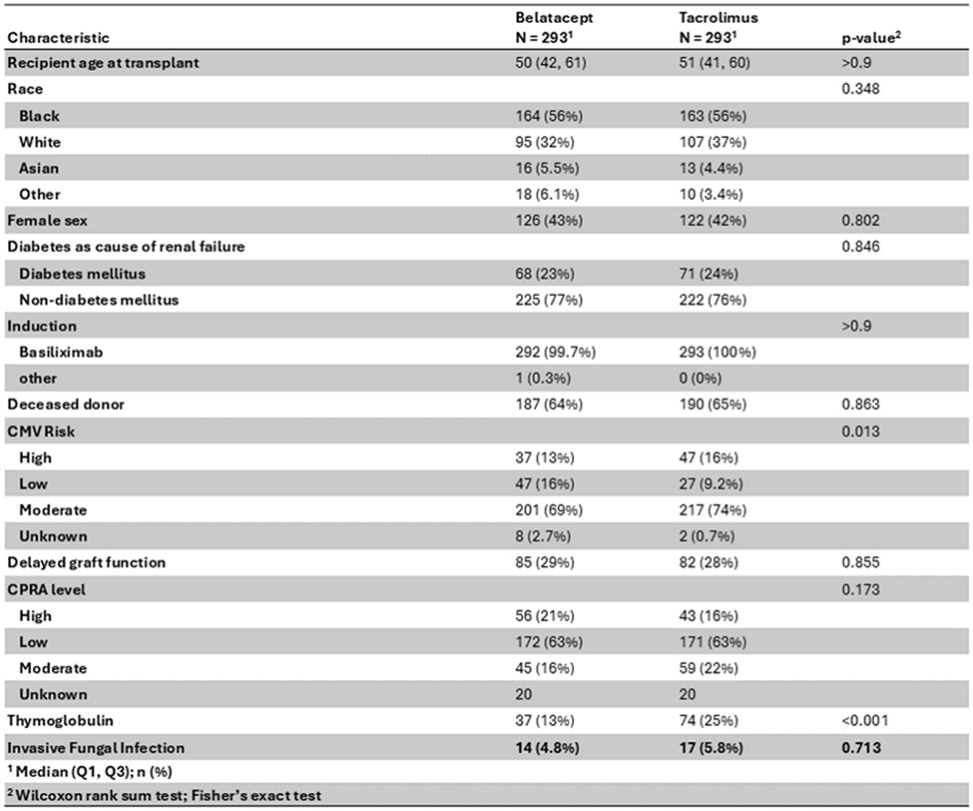
Clinical Characteristics of the Study Cohort

**Figure:**
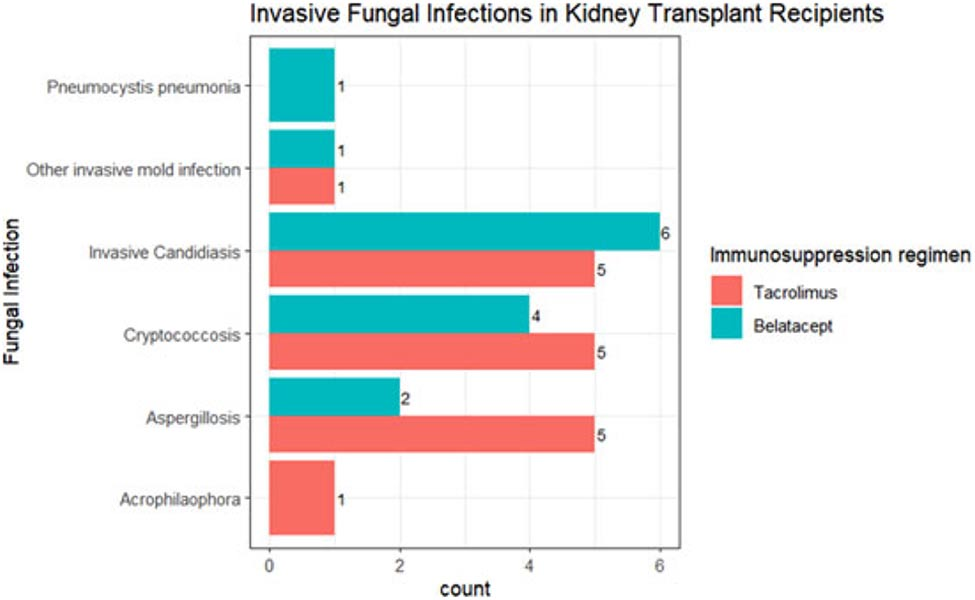
Invasive Fungal Infections in Kidney Transplant Recipients

**Methods:**

We conducted a retrospective propensity-matched study of adult HIV-negative KTR transplanted at our center between 2016-2020 who received de-novo belatacept- or tacrolimus-based immunosuppression. Matching variables included sex, donor type, age at transplant, HLA mismatch, and diabetes as cause of kidney failure. Data was extracted from the clinical data warehouse. IFI was identified via ICD codes and confirmed by chart review using EORTC/MSGERC definitions. Clinical characteristics were compared using Wilcoxon Rank and Fisher Exact tests. Time to IFI was evaluated with Kaplan Meier curves log-rank testing.

**Results:**

A total of 293 belatacept- and 293 tacrolimus-treated KTR were compared. Proven IFI occurred in 14 (4.8%) belatacept- and 17 (5.8%) tacrolimus-treated KTR (p=0.713, Table). Median time to IFI was 370 days (IQR 122, 555) vs 359-days (IQR 225,979), respectively (p=0.48). Event rates were 1.09 per 100-person years in both groups. IFI in belatacept patients included invasive candidiasis (N=6), cryptococcosis (N=4), invasive aspergillosis (N=2), *Pneumocystis* pneumonia (N=1), and non-speciated mold causing invasive fungal sinusitis (N=1). IFI in the tacrolimus group included invasive candidiasis (N=5), invasive aspergillosis (N=5, including 1 with *Paecilomyces* coinfection), cryptococcosis (N=5), *Acrophilaophora* brain abscess (N=1), and non-speciated mold causing invasive sinopulmonary fungal infection (N=1) (Figure). Death occurred in 3/14 (21%) belatacept vs 8/17 (47.1%) tacrolimus patients with IFI (p=0.258).

**Conclusion:**

De-novo belatacept-based immunosuppression was not associated with increased risk of IFI compared to tacrolimus. These findings suggest routine antifungal prophylaxis is not indicated in KTR receiving de-novo belatacept-based immunosuppression.

**Disclosures:**

Christian P. Larsen, MD, PhD, Bristol-Myers Squibb: Advisor/Consultant|CareDx: Advisor/Consultant|Eledon: Advisor/Consultant

